# Preparation of PbS Nanoparticles by Phase-Transfer Method and Application to Pb^2+^-Selective Electrode Based on PVC Membrane

**DOI:** 10.1080/00032710802421780

**Published:** 2008-12-02

**Authors:** Weihong Song, Chunhui Wu, Hongzong Yin, Xiaoyan Liu, Panpan Sa, Jinyang Hu

**Affiliations:** College of Chemistry and Material Science, Shandong Agricultural University, Shandong, P. R. China

**Keywords:** Ion-selective electrode, lead sensors, PbS nanoparticles, phase-transfer method, PVC membrane

## Abstract

A novel approach to prepare homogeneous PbS nanoparticles by phase-transfer method was developed. The preparatory conditions were studied in detail, and the nanoparticles were characterized by transmission electron microscopy (TEM) and UV-vis spectroscopy. Then a novel lead ion-selective electrode of polyvinyl chloride (PVC) membrane based on these lead sulfide nanoparticles was prepared, and the optimum ratio of components in the membrane was determined. The results indicated that the sensor exhibited a wide concentration range of 1.0×10^−5^ to 1.0×10^−2^ mol.L^−1^. The response time of the electrode was about 10 s, and the optimal pH in which the electrode could be used was from 3.0 to 7.0. Selectivity coefficients indicated that the electrode was selective to the primary ion over the interfering ion. The electrode can be used for at least 3 months without any divergence in potential. It was successfully applied to directly determine lead ions in solution and used as an indicator electrode in potentiometric titration of lead ions with EDTA.

## INTRODUCTION

Lead is ubiquitous in the environment. In recent years, because of the increasing use of lead and its serious hazardous effect to human health, much effort has been placed on the development of ion-selective electrodes (ISEs) for detecting of lead ions. Most of ISEs are polyvinyl chloride (PVC) membrane electrodes. Their dynamic response is generated by dispersing different active material as an ion carrier in a PVC matrix.

A number of diverse ligands, viz., *N*′-dibenzyl-1,4,10,13-tetraoxa-7,16-diazacyclooctadecane (Gupta, Jain, and Kumar 2006), N,N′-bis(salicylidene)-2,6-pyridinediamine (Jeong et al. 2005), *meso*-tetrakis-(2-hydroxy-1-naphthyl) porphyrin (Ardakany 2004), capric acid (Mousavi, Barzegar and Sahari 2004), benzyl disulphide (Abbaspour and Tavakol 1999), 5,5′-dithiobis-(2-nitrobenzoic acid) (Rouhollahi, Ganjali, and Shamsipur 1998), tetrabenzyl pyrophosphate (Xu and Katsu 2000), 9,10-anthraquinone derivative (Tavakkoli 1998), and 3,4,4a,5-tetrahydro-3-methylpyrimido-[1,6-a] benzimidazole-1(2H) thione (Jain, Sondhi, and Rajvanshi 2003) were used to prepare Pb^2+^ sensors. Besides these, crown ethers and calixarenes were also widely investigated as sensing materials, including benzo-15-crown-5 (Sheen and Shih 1992), diaza-crown ether (Yang et al. 1997), 18-crown-6 (Zareh, Ghoneom, and El-Aziz 2001), N,N′-dimethylcyanodiaza-18-crown-6 (Ganjali 2002), *sym*-dibenzo-16-crown-5 ethers (Su, Chang, and Liu 2001), 4′-vinylbenzo-15-crown-5 homopolymer (Ganjali et al. 1998), lariat crown ethers (Hasse 2001), 1,10-dibenzyl-1,10-diaza-18-crown-6 (Mousavi 2000), thia-crown ether (Shamsipur, Ganjali, and Rouhollahi 2001), calix[*n*] arene phosphine oxide derivatives (Cadogan 1999), di- and tetrathioamide calyx[4]arene (Malinowska 1994), thiophosphorylated calyx[6]arene (Wroblewski 1996), 4-*tert*-butylcalix[6]arene (Bhat, Ijeri, and Srivastava 2004), calixarene carboxyphenyl azo derivative (Lu, Chen, and He 2002), 2,12-dimethyl-7,17-diphenyltetrapyrazole and 5,11-dibromo-25,27-dipropoxy-calix[4]arene (Jain et al. 2006), and 4-*tert*-butylcalix[4]-arene (Gupta, Mangla, and Agarwal 2002). In addition, nanoparticles used as ionophores have ever been reported, such as nanosized PbO powders (Li et al. 2005). However, a lead ion-selective electrode based on homogeneously nanosized PbS particles has never been reported.

In this work, a novel approach to prepare homogeneous PbS nano-particles by a phase-transfer method is first described, followed by characterization of the nanoparticles TEM and UV-vis spectra. Performances of lead ion-selective PVC electrode based on PbS nanoparticles prepared were also studied. The results, reported in the present communication, showed that this sensor based on PbS nanoparticles exhibited good sensitivity and selectivity toward lead ions and could therefore be used as a selective sensor for its quantification.

## EXPERIMENTAL

### Reagents

High-molecular-weight polyvinylchloride (PVC) and dioctylphthalate (DOP) were obtained from Aldrich. Analytical reagent-grade tetrahydrofuran (THF), nitric acid, and sodium hydroxide were obtained from Shanghai Chemical Reagent Corporation. Solutions of metal (nitrates) were prepared in doubly distilled water and standardized by the reported methods whereever necessary. Working solutions of different concentrations were prepared by diluting 0.1 mol.L^−1^ stock solutions.

### Apparatus

The microstructure and morphology of the nanoparticles were characterized by TEM (model 800, Hitachi) and UV-vis spectrophotometry (model UV-1601PC, Shimadzu), respectively. The potential measurements were carried out with a digital pH meter (model pHS-3D).

### Preparation of PbS Nanoparticles

A 60-mL portion of 1 × 10^−4^ mol·L^−1^ lead acetate was placed in a beaker and adjusted to pH 6.0 with 1 mol·L^−1^ sodium hydroxide under continuous stirring. The solution was then transferred to a 150-mL separatory funnel. Then 60 mL of 1.5 × 10^−4^ mol·L^−1^ H_2_ Dz/CCl_4_ solution was added, and the mixed solution was oscillated thoroughly for 20 min. The color of the organic phase changed from deep green to pink, proving that lead ions were transferred from water phase to the organic phase by extraction. The two phases were stratified thoroughly after 2 h, and the transparent organic phase was transferred to an Erlenmeyer flask. Then 60 mL of 1 × 10^−4^ mol·L^−1^ CH_3_CSNH_2_/CH_3_CH_2_OH solution was added slowly in a dropwise fashion into the flask under vigorous stirring, while the dropping speed was controlled to about 10 drops per minute. The mixed solution was stirring for 12 h at room temperature, and then the PbS nanoparticles formed in the solution.

### Preparation of Nano-PbS-PVC Membranes

An amount of 0.1000 g of PVC powder was put into a 100-mL beaker and dissolved in 10 mL of THF. Then 0.1000 g of DOP was added and mixed homogeneously. Then the solution was put into a Petri dish (90 mm in diameter and 18 mm in height). Fifty mL of solution containing PbS nanoparticles was also added. The resulting solution was left overnight at room temperature for evaporation of the organic solvent. A transparent membrane of about 0.2 mm in thickness was obtained.

### Preparation of PbS-PVC Membrane Electrode

**Figure 1:**
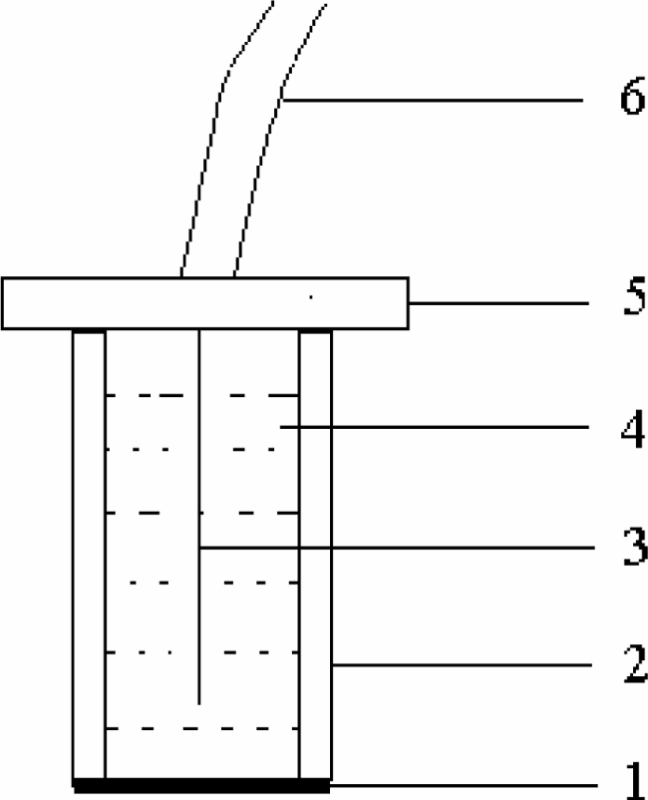
Structure of PbS-PVC membrane electrode: 1, sensitivity membrane; 2, PVC tube; 3, Ag/AgCl coated wire internal reference electrode; 4, internal solution; 5, electrode base; and 6, electrode wire.

A 100-mm PVC tube (10 mm in diameter) was used as electrode body. An 80-mm Ag/AgCl wire was fixed in the tube and acted as an internal reference electrode. Then a disk of about 12 mm diameter was cut from the obtained membrane. The disk was glued to the polished end of a PVC tube by THF. The tube was then filled with internal solution [0.01 mol·L^−1^ Pb(NO_3_)_2_]. The structure of PbS-PVC membrane electrode is shown in Fig. 1. The electrode was finally equilibrated for 24 h in a 1 × 10^−7^ mol·L^−1^ Pb^2+^ solution for performance measurements.

### Potential Meaurements

A cell assembly of the following type was used:

$$\begin{array}{l} \text{Ag}/\text{AgCl}|0.01\text{mol}\cdot {\text{L}}^{-1}{\text{Pb}}^{2+},\text{ pH 6 PVC membrane}\\ \|\text{test solution},\text{pH}6|\text{Ag}/\text{AgCl reference electrode} \end{array}$$

The electrodes were immersed directly in the test solution at 25 ± 0.2°C. The electric potential of lead acetate standard solutions (with concentrations from 1×10^−7^ to 1 × 10^−1^ mol·L^−1^) were measured by a pHS-3D potentiometer.

All pHadjustments were made with diluted solutions of HNO_3_ or NaOH.

## RESULTS AND DISCUSSION

### Characterization of Prepared PbS Nanoparticles

**Figure 2:**
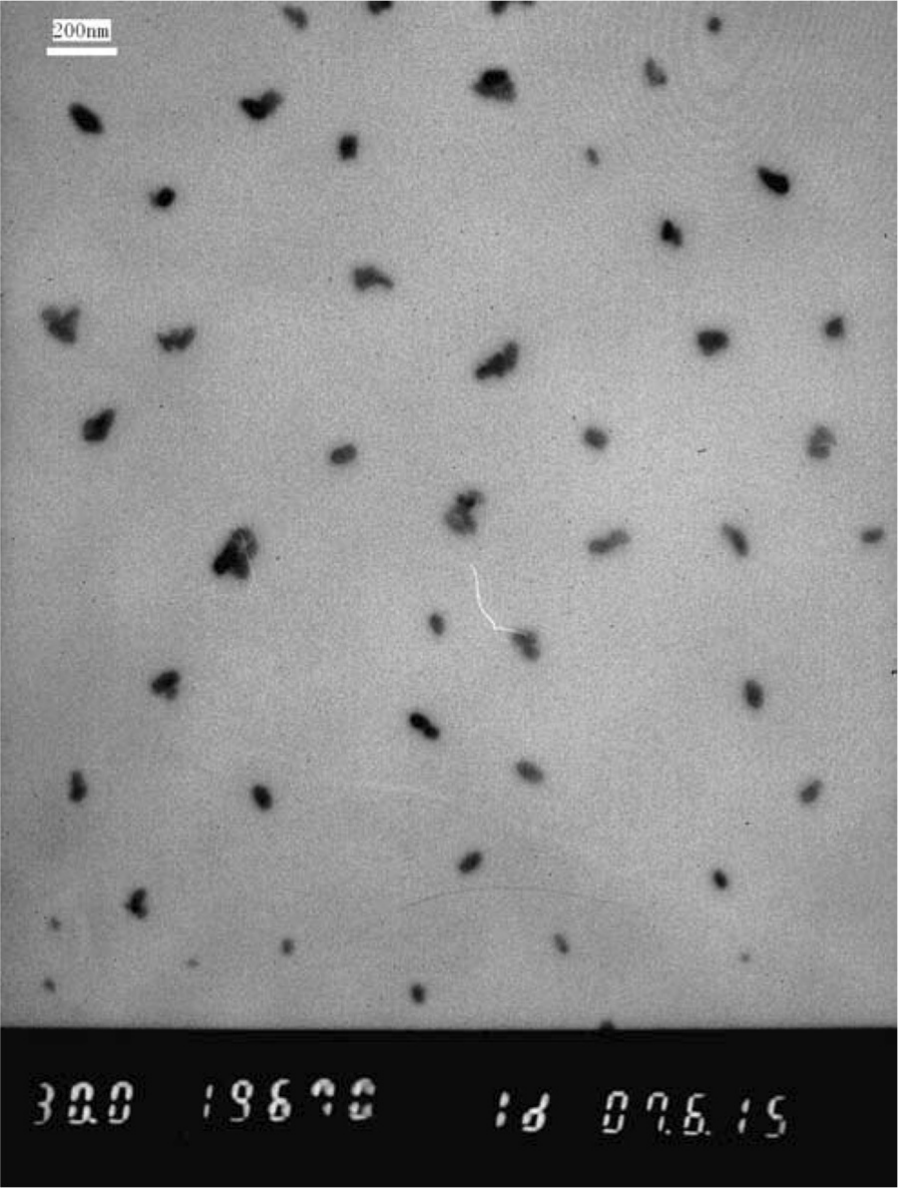
TEM image of PbS nanoparticles.

TEM has been used rather extensively to measure the nanoparticle size in a direct and visual manner. Figure 2 shows the TEM micrographs of PbS nanoparticles. The majority of the particles' sizes fall into the range of 40 to 60 nm in diameter. It is noted that most of the particles have compact spherical structures with proportional size distribution. Although some portion aggregates during the sample preparation, the overall distribution is still very well. The nanoparticles can be stored from 3 to 5 days at room temperature without any obvious aggregation.

**Figure 3:**
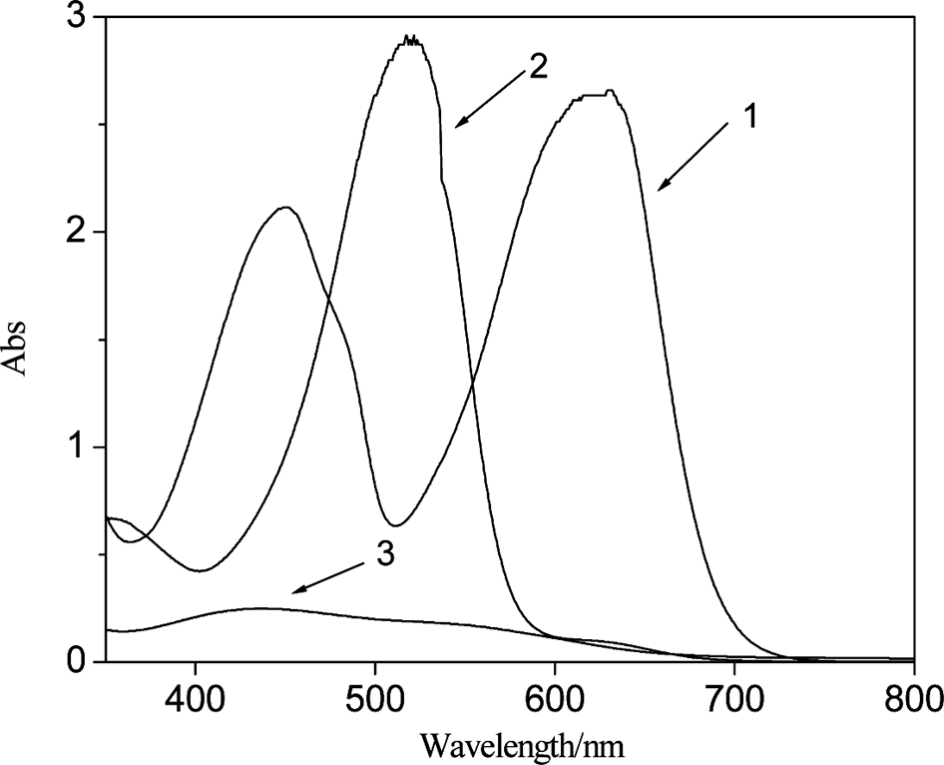
UV-vis spectrum of Pbs nanoparticles: 1, H_2_D_z_/CCl_4_; 2, Pb^2+^ – H_2_D_z_/CCl_4_; 3, PbS nanoparticles.

Nanosized particles generally exhibit threshold energy in the optical absorption measurements because of the size-specific band gap structures (Wroblewski et al. 1996; Bhat, Ijeri, and Srivastava 2004; Lu, Chen, and He 2002; Jain et al. 2006), which is reflected by the blue shifting of the absorption edge (from near-infrared to visible) with decreasing particle size (Chen, Templeton, and Murray 2000; Steigerwald and Brus 1990; Weller 1993; Zhang 1997; Borrelli and Smith 1994; Fendler 1987. From the optical spectra of PbS nanoparticles (Fig. 3), it is seen that the H_2_D_z_/CCl_4_ solution and Pb^2+^ –H_2_D_z_/CCl_4_ solution showed rather large absorption band at 629 nm and 517 nm, respectively. Otherwise, the PbS nanoparticles showed a broad but not strong peak at 437 nm and obvious blue shifting, which confirmed the production and remarkable quantum effect of PbS nanoparticles.

### The Influencing Factors of Nano-PbS Preparation

The concentration of lead ions was a key factor to the prepared PbSnanoparticles. If the solution contained a high concentration of Pb^2+^ ions, the reaction speed with sulfide ions was too fast to distribute in time, resulting in that PbS nanoparticles that easily aggregated. In contrast, if the concentration of Pb^2+^ ions solution was low, the resulting PbS particles would be small. The response of nano-PbS-PVC membrane electrodes had poor performances. Therefore, 1 × 10^−4^ mol·L^−1^ lead ion was adopted.

**Figure 4:**
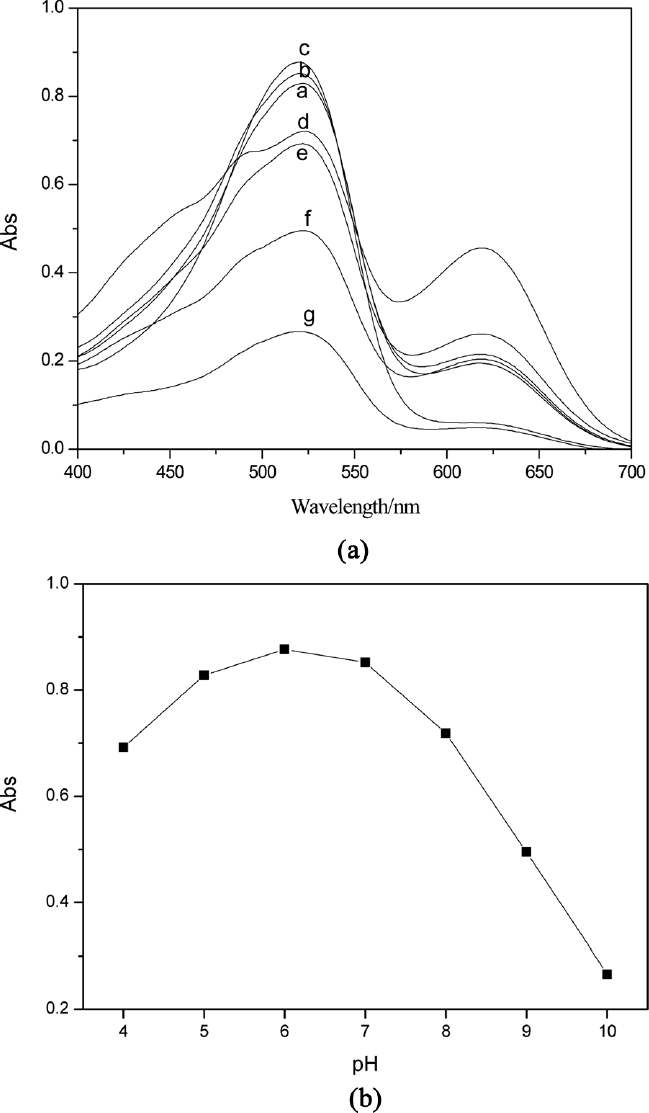
Variation of UV-vis spectrum with different acidity of Pb^2+^ ion solution: a = 4; b = 5;c = 6; d = 7; e = 8; f = 9; and g = 10.

The influence of the acidity on concentration of Pb^2+^ after 20 min of extraction was investigated. The UV-vis spectrum of Pb^2+^ –H_2_D_z_/CCl_4_solution was shown in Fig. 4a. The results showed that the H_2_D_z_ could extract Pb^2+^ from the water phase to the CCl_4_ organic phase in pH 4 to 10. The maximum intensity of absorption at 517 nm is shown in Fig. 4b. The optimal pH value was 6. The solution of PbS nanoparticles prepared at this pH had good stability and could be stored for a long time.

**Figure 5:**
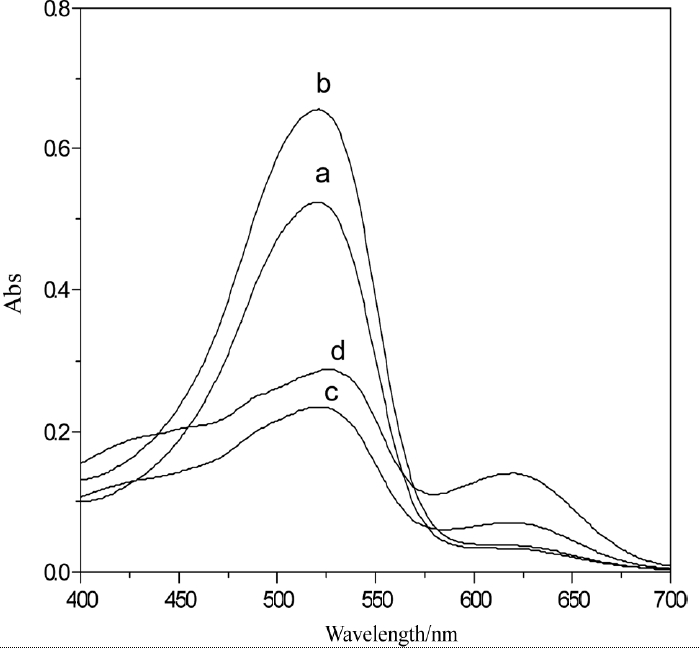
Influence of the ratio between H_2_D_z_ and CCl_4_: a = 1:1; b = 1:1.5; c = 1:2; and d = 1:2.5.

The influence of the ratio between H_2_D_z_ and CCl_4_ was also investigated, as illustrated in Fig. 5. The optimal ratio was 1:1.5. Considering the solubility of H_2_D_z_ in CCl_4_ and the concentration of Pb^2+^ ions, 1.5 × 10^−4^ mol·L^−1^ H_2_D_z_/CCl_4_ solution was used.

Three methods were used to obtain sulfide ions: Na_2_S/H_2_O solution, CH_3_CSNH_2_/H_2_O solution, and CH_3_CSNH_2_/CH_3_CH_2_OH solution. When the Pb^2+^–H_2_D_z_/CCl_4_ solution was mixed with Na_2_S/H_2_O or CH_3_CSNH_2_/H_2_O solution, two phases were formed. The reaction only took place at the interface. The sulfide ions in water were so bulky that plenty of large PbS particles were produced. Otherwise, the CH_3_CSNH_2_/CH_3_CH_2_OH solution could be mixed thoroughly with Pb(□) H_2_Dz/CCl_4_ solution.The reaction, therefore, occurred in the homogeneous phase. Furthermore, the speed of sulfide ions released from thioacetamide in anhydrous ethanol was not fast, and thus homogeneous PbS nanoparticles could be obtained. The TEM images showed that these nanoparticles had small diameters and well-proportioned size distribution. The optimal concentration of thioacetamide in anhydrous ethanol was 1.0 10 4 mol L 1 from the experimental results.

### The Composition of Membrane Electrode

Membrane composition was investigated to evaluate the performance of the lead ion-selective electrode based on nano-PbS-PVC membrane. The DOP was a plasticizer that could increase the membrane flexibility. Without DOP, the membrane would turn hard and fragile. As a result, the electrode had no response signal and lead ion. With too much DOP, the membrane would be viscid and its mechanical intensity would decrease. When the mass of PVC powder to DOP was 0.1000 g respectively, a 0.2-mm-thick membrane was formed after THF evaporated. The membrane had appropriate thickness, good flexibility, and high mechanical intensity. It was found that the thickness of 0.2 mm was optimal through the experiments. The performance of the ion-selective electrode developed was excellent.

### Effect of Internal Solution

**Figure 6:**
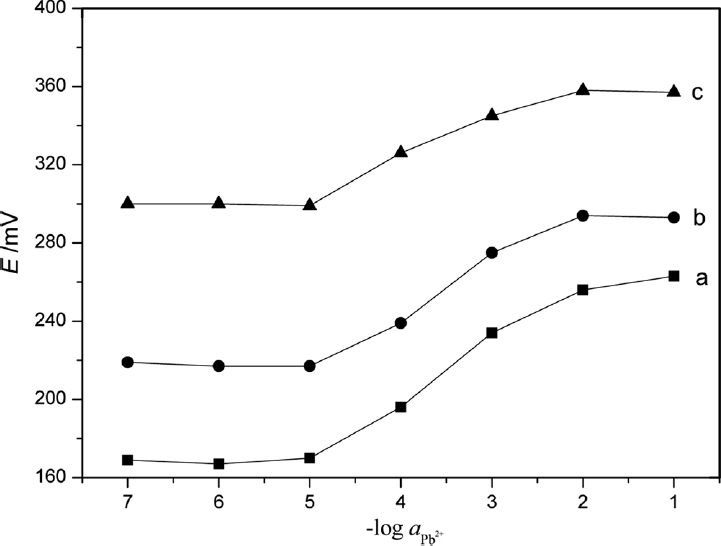
Influence of the potential response of Pb^2+^ selective electrode with different internal filling solution: a, 1 × 10^−2^ mol·L^−1^; b, 5 × 10^−2^ mol·L^−1^; and c, 1 × 10^−1^ mol·L^−1^.

In accord with the responses of the generally adopted ion sensor, the internal solution could affect the sensor response when the membrane internal diffusion potential was appreciable. Thus, the effect of activity of the internal solution on the functioning of the membrane sensors was studied by measuring the potentials at varying activity of internal solution, viz. 1.0 × 10^−2^, 5.0 × 10^−2^, and 1.0 × 10^−1^ mol·L^−1^ Pb^2+^ (Fig. 6). Best results in terms of slope and working concentration range were obtained with internal solution of activity 1.0 × 10^−2^ mol·L^−1^. Thus, the activity of the internal solution was kept at 1.0 × 10^−2^ mol·L^−1^ in all studies.

### The Influence of the Content of PbS Nanoparticles

**Figure 7:**
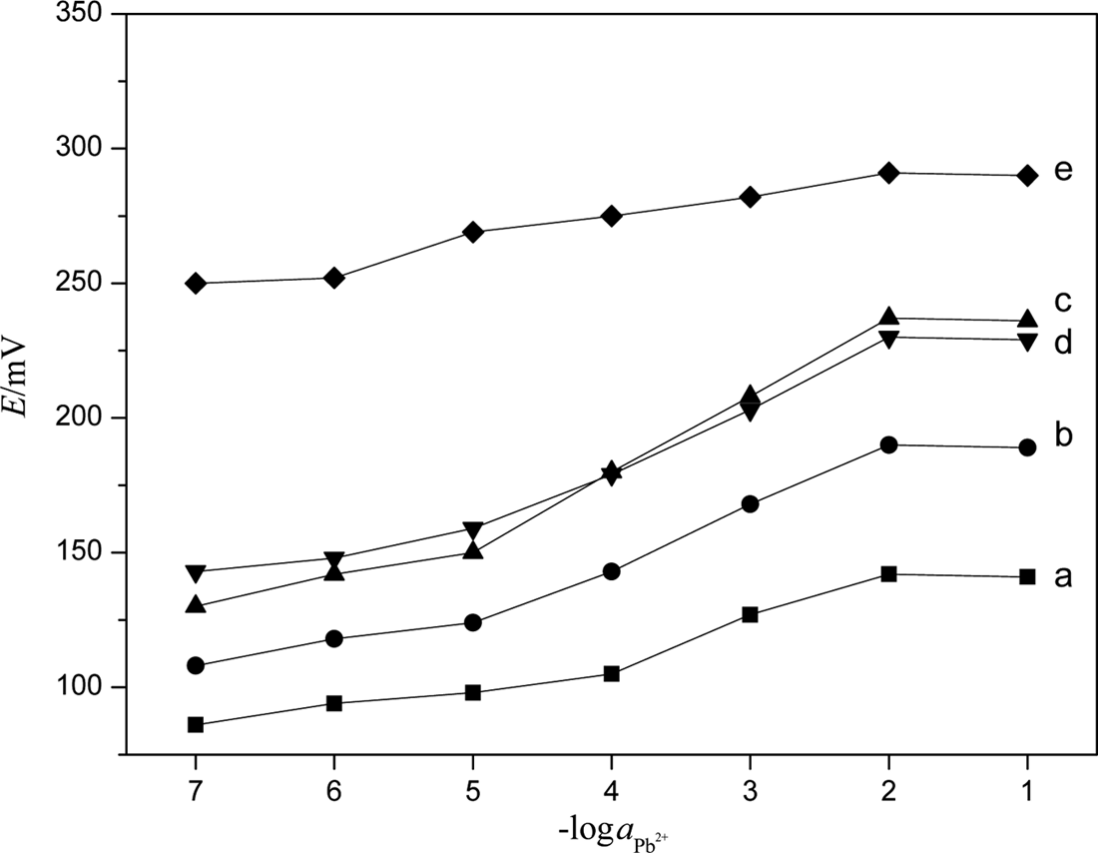
Variation of the potential of PVC membrane with different volume of PbS nanoparticles: a, 10 mL; b, 20 mL; c, 30 mL; d, 40 mL; and e, 50 mL.

As an active component in the membrane, nano-PbS directly affected the electrode performance in a dose-dependent manner. Various volumes of PbS nanoparticles, viz. 10, 20, 30, 40, 50, and 60 mL of solution containing PbS nanoparticles, were added to prepare six membranes. The performances of those electrodes were tested in a series of lead ion standard solutions from 1.0 × 10^−7^ to 1.0 × 10^−1^ mol·L^−1^. It was seen from Fig. 7 that the electrode made of membrane containing 30 or 40 mL of PbS nanoparticle solution had better performance.

### pH Effect of Test Solution

**Figure 8:**
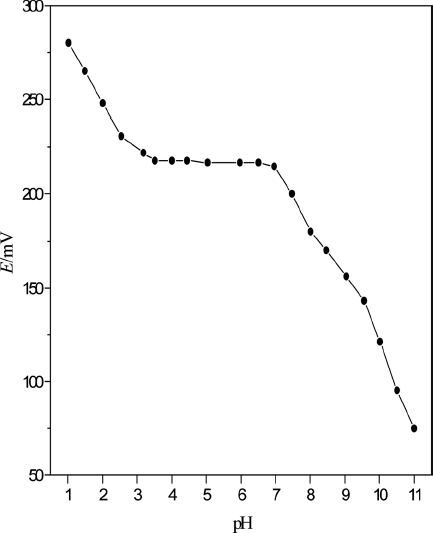
Effect of pH on cell potential of sensors at Pb^2+^ activity of 1.0 × 10^−3^ mol·L^−1^.

With 40 mL of PbS nanoparticles as ionophores and DOP as a plasticizer, the response of Pb^2+^ selective electrode affected by pH was studied (Fig. 8). The pH value of 10 × 3 mol·L^−1^ Pb(NO_3_)_2_ test solutions was adjusted with HNO_3_ and NaOH. As illustrated in Fig. 8, the potentials remained constant when the pH value was kept from 3.0 to 7.0. Outside this range, the electrode response changed drastically. This was probably due to lead hydroxide formation and response of the electrode to hydrogen ions at low pH values.

### Response Time and Lifetime

The response time of the electrodes was found to be approximately 10 s when the electrodes were directly immersed in 10^−3^ or 10^−4^ mol·L^−1^solution; the response times of the membrane electrodes were 6 and 8 s, respectively. Even if the concentration of Pb^2+^ ions was 10^−5^ mol·L^−1^, the response time would also be stable within 15 s.

The lifetime of the electrodes was determined by recording its potential at an optimum pH value and plotting its calibration curve each day. The parameters, such as the slope, working range, and response time of the electrode, were found to be reproducible. Also, the membrane could be used over a period of 3 months without observing any significant drift in the parameters. After this period, a slight deviation was observed in response time and slope, which could be corrected by re-equilibrating the membrane with 1.0 × 10^−7^ mol·L^−1^ lead ion solution for 2–3 days. With this treatment, the assembly could be used over a period of about 1 more month and then it was replaced by a fresh membrane.

### Potentiometric Selectivity

**Figure 9:**
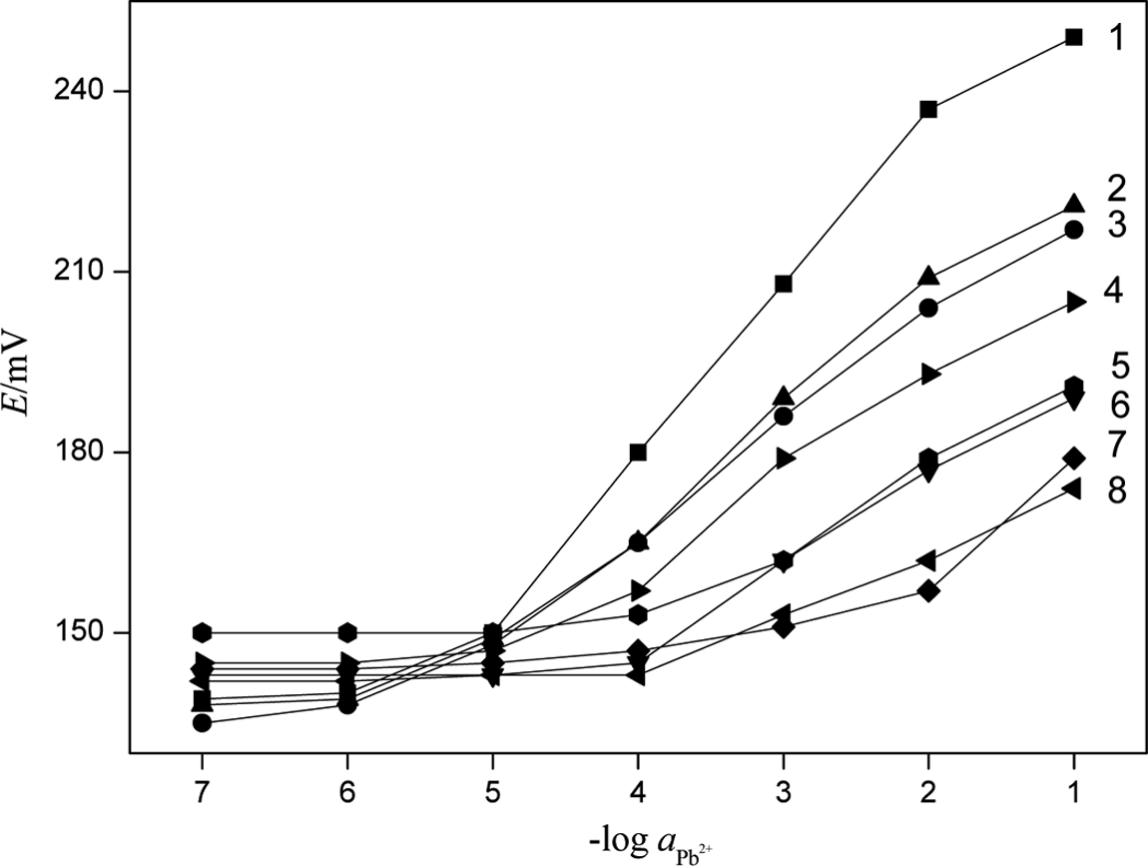
The response to some cations of Pb^2+^ selective electrode based on PbS nanoparticles: 1, Pb^2+^; 2, Na^+^; 3, K^+^; 4, Ca^2+^; 5, Ba^2+^; 6, Cu^2+^; 7, Al^3+^; and 8, Ni^2+^.

The modified fixed interference method as suggested by Viteri and Diamond (1994) was used with 1.0 × 10^−2^ mol L ^−1^ interfering ions to determine selectivity coefficients of the proposed sensor. Selectivity parameter data for various ions are presented in Fig. 9 and Table 1. A value of selectivity coefficient less than 1 indicates that the electrode was selective to the primary ion over the interfering ion. However, it was important to mention that the smaller value of selectivity coefficient was, the higher selectivity of the electrode. In this respect, the selectivity coefficients for K^+^ and Na^+^ are not very small. Although the sensor is selective even over these two ions, the order of selectivity is not very high. It suggested that lower concentrations of K^+^ and Na^+^ would not cause interference, but higher levels would cause interference.

**Table 1:** Selectivity coefficients ($K_{{\text{pb}}^{+2},\text{j}}^{\text{pot}\prime }$) obtained in the presence of various interfering ions for the lead electrode

Interfering ion	$K_{{\text{pb}}^{+2},\text{j}}^{\text{pot}}$
K^+^	7.2 × 10^−1^
Na^+^	6.7 × 10^−1^
Cu^2+^	9.94 × 10^−1^
Ca^2+^	1.25 × 10^−2^
Ba^2+^	1.31 × 10^−2^
Ni^2+^	1.17 × 10^−2^
Al^3+^	5.25 × 10^−3^

## ANALYTICAL APPLICATIONS

**Figure 10:**
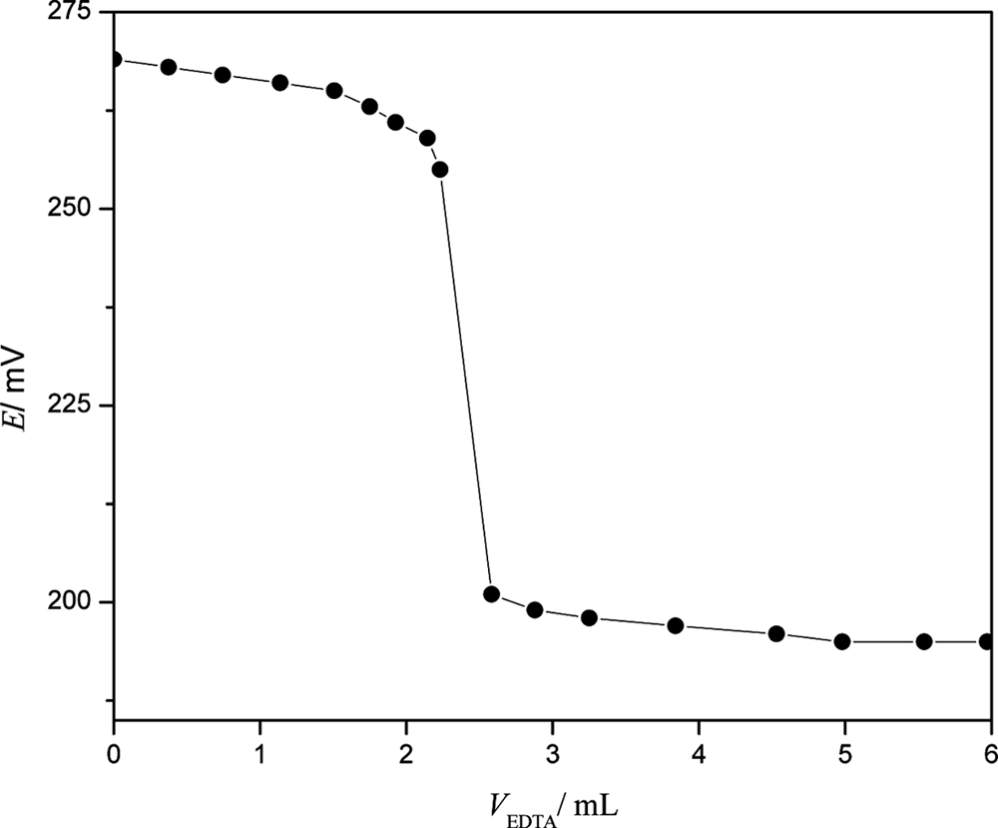
Potentiometric titration plot of 1.0 × 10^−4^ mol. L ^−1^ Pb^2+^ solution (25 mL) with 1.0 × 10^−3^ mol·L^−1^ EDTA solution.

The analytical application of the electrode was investigated as an indicator electrode in the potentiometric estimation of Pb^2+^ solution by titrating 25 mL of 1.0 × 10^−4^ mol L^−1^ Pb(NO_3_)_2_ against 1.0 × 10^−3^ mol·L ^−1^ EDTA solution. The pH of the solution was maintained at 6.0 throughout the titration with HNO_3_ and NaOH. The titration plot does not have a standard sigmoid shape (Fig. 10), which may be due to some interference caused by Na^+^ ions of the disodium EDTA salt (Gupta and Kumar 1999; Gupta et al. 1999). However, the sharp breakpoint corresponded to the stoichiometry of Pb^2+^ -EDTA complex show the efficacy of the proposed electrode in the potentiometric estimation of Pb(II).

## CONCLUSIONS

In this work, homogeneous lead sulfide nanoparticles were successfully prepared by the phase-transfer method. TEM and UV-vis spectroscopy showed that PbS nanoparticles had good spherical structure with proportional size distribution, which could be used as ion carriers in the development of lead ion-selective electrode. The sensor prepared by PbS nanoparticles exhibited good reproducibility and fast response time and could be employed for more than 3 months. It could also be used as an indicator electrode in the potentiometric titration of lead ions with EDTA. Otherwise, the results of potentiometric selectivity indicated that most of metal ions would not affect the selectivity of the lead electrode seriously. Therefore, the proposed sensor is a good addition to the existing list of the lead ion-selective sensors reported and can be used for real sample analysis.
